# DEVELOPMENT AND VALIDATION OF THE NOVEL MEDICATION TAKING INSTRUMENT - FOUR FACETS OF ADHERENCE (MTI-4)

**DOI:** 10.1093/geroni/igad104.3272

**Published:** 2023-12-21

**Authors:** Candace Ryan, Debra Schutte, Ana Daugherty

**Affiliations:** Wayne State University, Detroit, Michigan, United States; Wayne State University, Detroit, Michigan, United States; Wayne State University, Detroit, Michigan, United States

## Abstract

Medication adherence is critical for disease management among aging African-Americans who are at greatest risk for poor adherence and affliction with chronic diseases. However, medication adherence is a complex behavior with distinct facets (frequency, pattern of adherence, barriers, and beliefs related to adherence), which current instruments cannot comprehensively measure in this underrepresented population. The Medication Taking Instrument – Four Facets of Adherence is an instrument developed to measure all facets of adherence among diverse, community-dwelling aging adults using the innovative application of the “Nursing Rights” of medication administration. Items were developed following a scoping review of the literature. Validation was obtained from medical professionals in Canada and the United States who prescribe medications to diverse patient populations. 12 of 13 medical professionals responded to the survey request (67% Nurse Practitioners, 33% Physicians) and rated each item (0=Low Agreement to 5=High Agreement) for clarity and validity of each item representing the specific facet. Most instrument items obtained high agreement (response rating > 3) for both validity and clarity from 83% or more of respondents. Experts’ feedback augmented items for two facets (barriers and beliefs), improved clarity, minimized jargon, and supported instrument use in the clinical setting. The resulting instrument will comprehensively characterize medication adherence, direct more targeted research aims, and guide interventions to augment adherence among aging African Americans. Ongoing data collection in the Detroit Aging Brain Study will provide additional tests of factor structure, internal scale reliability, and convergent validity using other indicators of medication history and symptom control.

